# Melatonin Ameliorates the Progression of Atherosclerosis via Mitophagy Activation and NLRP3 Inflammasome Inhibition

**DOI:** 10.1155/2018/9286458

**Published:** 2018-09-04

**Authors:** Sai Ma, Jiangwei Chen, Jing Feng, Ran Zhang, Miaomiao Fan, Dong Han, Xiang Li, Congye Li, Jun Ren, Yabin Wang, Feng Cao

**Affiliations:** ^1^Department of Cardiology & National Clinical Research Center of Geriatrics Disease, Chinese PLA General Hospital, Beijing 100853, China; ^2^Department of Cardiology, Xijing Hospital, Fourth Military Medical University, Xi'an 710032, China; ^3^Department of Emergency Medicine, Jinling Hospital, Nanjing 210000, China; ^4^College of Health Sciences, University of Wyoming, Laramie, WY 82071, USA

## Abstract

The NLRP3 (nucleotide-binding domain and leucine-rich repeat pyrin domain containing 3) inflammasome-mediated inflammatory responses are critically involved in the progression of atherosclerosis (AS), which is the essential cause for cardiovascular diseases. Melatonin has anti-inflammatory properties. However, little is known about the potential effects of melatonin in the pathological process of AS. Herein, we demonstrate that melatonin suppressed prolonged NLRP3 inflammasome activation in atherosclerotic lesions by reactive oxygen species (ROS) scavenging via mitophagy in macrophages. The atherosclerotic mouse model was induced with a high-fat diet using ApoE^−/−^ mice. Melatonin treatment markedly attenuated AS plaque size and vulnerability. Furthermore, melatonin decreased NLRP3 inflammasome activation and the consequent IL-1*β* secretion within atherosclerotic lesions. Despite the unchanged protein expression, the silent information regulator 3 (Sirt3) activity was elevated in the atherosclerotic lesions in melatonin-treated mice. In ox-LDL-treated macrophages, melatonin attenuated the NLRP3 inflammasome activation and the inflammatory factors secretion, while this protective effect was abolished by either Sirt3 silence or autophagy inhibitor 3-MA. Mitochondrial ROS (mitoROS), which was a recognized inducer for NLRP3 inflammasome, was attenuated by melatonin through the induction of mitophagy. Both Sirt3-siRNA and autophagy inhibitor 3-MA partially abolished the beneficial effects of melatonin on mitoROS clearance and NLRP3 inflammasome activation, indicating the crucial role of Sirt3-mediated mitophagy. Furthermore, we demonstrated that melatonin protected against AS via the Sirt3/FOXO3a/Parkin signaling pathway. In conclusion, the current study demonstrated that melatonin prevented atherosclerotic progression, at least in part, via inducing mitophagy and attenuating NLRP3 inflammasome activation, which was mediated by the Sirt3/FOXO3a/Parkin signaling pathway. Collectively, our study provides insight into melatonin as a new target for therapeutic intervention for AS.

## 1. Introduction

Atherosclerosis (AS), with a high rate of morbidity and mortality, is a predominant cause for cardiovascular diseases. Also, AS has been identified as a chronic inflammatory disease, in which inflammation is a primary driving force for lesion rupture [1]. Inflammasomes are cellular protein complexes that could respond to cellular damage, whose formation requires the pattern recognition receptor (PRR). NLRP3 (nucleotide-binding domain and leucine-rich repeat pyrin domain containing 3) a recognized type of PRR, is a cytoplasmic receptor responding to danger signals. Upon activation, NLRP3 interacts with adapter apoptosis-associated speck-like protein containing a C-terminal caspase recruitment domain (ASC) to form NLRP3 inflammasome, leading to caspase-1 activation and the release of proinflammatory cytokines [2, 3]. Since the NLRP3 inflammasome is considered a cellular machinery involved in the activation of inflammatory processes, it is necessary to study its role in the progression of atherosclerotic plaque.

Melatonin (N-acetyl-5-methoxytryptamine (MEL)), a predominant indoleamine of the pineal gland with marked antioxidant, antiageing, and metabolic properties, has been reported to exert beneficial effects on cardiovascular diseases including myocardial ischemia-reperfusion, hypertension, and heart failure [4–9]. Recently, the protective signaling pathways of melatonin have attracted more and more attention. One of the essential mechanisms mediating the protective of melatonin is via the activation of silent information regulator 3 (Sirt3). Yu et al. illustrated melatonin ameliorated cardiac ischemia/reperfusion injury through the activation of the Sirt3 axis [10]. However, the potential function of melatonin in AS is not completely understood [11]. Therefore, it is of great importance to demonstrate the underlying protective action of melatonin for its clinical application.

In the current study, we hypothesized that melatonin protects against AS via the regulation of NLRP3 inflammasome. The study designs are as follows: the atherosclerotic model was induced with a high-fat diet (HFD) in ApoE^−/−^ mice. Atherosclerotic plaque progression and stability were examined with histological and immunofluorescence staining. An *in vitro* study was performed in oxidized low-density lipoprotein- (ox-LDL-) treated macrophage cell line RAW264.7 cells. Furthermore, we examined NLRP3 inflammasome activation and inflammatory factor secretion was tested, along with mitochondrial ROS generation, autophagy, mitophagy indexes, and potential signaling pathways. Forkhead box O3a (FOXO3a), which is a downstream target of Sirt3, has the abilities to regulate the expressions of antioxidant-encoding genes [12–14]. To investigate the potential role of the Sirt3/FOXO3a signaling pathway in the experimental conditions, siRNAs targeting Sirt3 and FOXO3a were applied.

## 2. Material and Methods

### 2.1. Animals

ApoE^−/−^ mice (male, 8 weeks old), purchased from the Animal Center in the Fourth Military Medical University, were initially fed with a standard laboratory chow diet for one week. Thereafter, animals were randomly divided into three groups (*n* = 20 each). In the control (Con) group, mice were maintained with a standard laboratory chow diet (TD.88137, Harlan Laboratories Inc., Madison, WI). In the atherosclerosis (AS) group, mice were fed with a Western-type diet (containing 15% fat and 0.25% cholesterol) for 12 weeks as previously reported [15]. In the melatonin-treated atherosclerotic (AS + MEL) group, melatonin administration started 4 weeks after the beginning of the Western-type diet. Melatonin (Sigma-Aldrich, St. Louis, MO, USA) was initially dissolved in ethanol and diluted with sterile water (final ethanol concentration 0.5%). Then, melatonin was intraperitoneally injected (20 mg/kg/d) for continuous 28 days (dosage determined according to our previous study) [16]. Animals were given free access to food and water during the whole experimental process. After the whole experimental period, the mice were sacrificed by cervical dislocation under anesthesia. All procedures were performed in accordance with the US National Institutes of Health (National Institutes of Health Publication number 85-23, revised 1996). Experimental protocols and animal care methods were approved by the Fourth Military Medical University Committee on Animal Care (XJYYLL-2014251).

### 2.2. *In Vitro* Experimental Protocol

The ox-LDL-treated RAW264.7 cell model was used for *in vitro* studies according to our previous reports [15, 17]. Raw 264.7 macrophages were incubated with DMEM (Gibco, Grand Island, NY, USA) supplemented with 10% fetal bovine serum (MDgenics, St. Louis, MO, USA) and 1% penicillin-streptomycin. Macrophages were maintained in a 5% CO_2_ incubator at 37°C. ox-LDL treatment (ox-LDL, 50 mg/ml for 24 h, Shanghai Leuven Biological Technology, Shanghai, China) was coadministered with or without melatonin (initially dissolved in ethanol and diluted with culture medium to a final concentration of 10 *μ*mol/l) in macrophages.

### 2.3. Small Interfering RNA

The small interfering RNA (siRNA) targeting of Sirt3 and siRNA targeting FOXO3 were purchased from GenePharma Co. Ltd. (Shanghai, China). The sequence of the mouse siRNA was as follows: Sirt3, 5′-ACUCCCAUUCUUCUUUCAC-3′; FOXO3, 5′-UUCAGAGACGAGGGUCCAAACACUG-3′. Twenty-four hours after seeding, cells were transiently transfected with 100 nM siRNA per dish at 80% confluence using the lipofectamine 2000 (Invitrogen Life Technology, Carlsbad, CA, USA). The knockdown efficiency of the target proteins was measured with Western blot assay.

### 2.4. Cytokine Measurement

The blood or cell supernatant was collected to measure inflammatory factors using a commercial ELISA kit (Sen-Xiong Company, Shanghai, China) according to the manufacturer's instructions. Each sample was tested in triplicate to achieve accuracy.

### 2.5. Mitochondrial ROS Generation

The amount of mitochondrial ROS production was measured with a MitoSOX™ Red Mitochondrial Superoxide Indicator (Thermo Fisher, Waltham, MA, USA). Briefly, cells were incubated with the MitoSOX dye with the final concentration of 5 *μ*M for 30 min at 37°C. Cells were then washed with PBS three times and collected for flow cytometry analysis.

### 2.6. Measurement of Mitochondrial Membrane Potential (MMP)

MMP was measured using a commercially available JC-1 probe (5,5′,6,6′-tetrachloro-1,1′,3,3′-tetraethyl-imidacarbocyanine iodide, Beyotime, Beijing, China). In brief, JC-1 dye was dissolved according to the manufacturer's instructions. Macrophages were incubated with JC-1 dye for 30 min at 37°C. After that, cells were washed with PBS and collected for consequential flow cytometry analysis.

### 2.7. Western Blot Assay

The Western blot analysis was done as described previously [18]. In brief, the vessel tissue or cellular proteins were collected using RIPA solution and separated with the SDS-PAGE gels. The samples were then transferred to polyvinylidene difluoride membrane (Millipore, USA) and incubated overnight (4°C) with primary antibodies including Sirt3, NLRP3, ASC, caspase 1, caspase 1-pro, FOXO3, acetylated protein, Parkin, and GAPDH (Cell Signaling Technology, MA, USA, 1 : 1000 dilution). Membranes were then washed with TBST and incubated with the secondary antibodies for 1 h at 37°C. Finally, bands were visualized with the chemiluminescence detection kit (Thermo Electron Corp., Rockford, USA) and analyzed with the Imagelab software system (Bio-Rad, CA, USA).

### 2.8. Transmission Electron Microscopy (TEM)

The cultured macrophages were rinsed with ice-cold PBS and centrifuged at 1000 ×g for 5 min at room temperature, after which the supernatants were removed. Cell pellets were fixed with 2.5% glutaraldehyde in 0.1 M cacodylate buffer with pH 7.4 for at least 30 min at 4°C. After fixation, the specimens were thoroughly washed in 0.1 M cacodylate buffer and then postfixed with 1% osmium tetroxide for 1 h at room temperature. The specimens were dehydrated using a graded series of ethanol and embedded in Epon. Finally, 0.1 *μ*m thin sections were stained with uranyl-acetate/lead citrate and viewed in a TEM (JEM-1230, JEOL, Tokyo, Japan).

### 2.9. Sirt3 Activity Assay

The Sirt 3 activity was analyzed using a commercial kit (Sirt3 Activity Assay Kit, Abcam, Cambridge, MA, USA) according to the manufacturer's instructions. Briefly, the activity of Sirt3 was measured by the basic principle of changing a Sirt3 reaction into the activity of the peptidase. In order to measure the enzyme activity of Sirt3, the activity of NAD^+^-dependent histone deacetylase was measured under the existence of Trichostatin A, which is the powerful inhibitor of HDACs. The results were read using a microplate reader capable of measuring fluorescence at Ex/Em = 340–360/440–460 nm. The Sirt3 activity was presented as the relative Sirt3 activity value (per the Con group).

### 2.10. Tissue Collection and Morphological Staining

To examine the atherosclerotic model and therapeutic effects of melatonin, the total carotid artery and descending aorta were removed from mice after euthanasia, followed by several morphological staining protocols, including hematoxylin-eosin (H&E), Oil Red O, and Masson trichrome staining. The arteries were fixed in a 4% paraformaldehyde solution, embedded in paraffin, and sectioned at 5 mm for the following staining. The sections were stained with H&E or Masson trichrome staining, and images of the stained sections were visualized using a light microscope (Olympus, Japan). The sizes of the plaque areas were quantified by delineating plaque areas in 3-4 root H&E stained sections/per mouse, and the data are presented as the mean plaque area. The necrotic core area was defined as the area that was negative for H&E staining within each plaque.

### 2.11. Immunofluorescence Microscopy

For mitochondrial staining, macrophages were incubated with MitoTracker® Red (Thermo Fisher, Waltham, MA, USA) at 37°C for 20 min. Then, cell slides or frozen tissue samples were fixed with 4% paraformaldehyde for 15 min at 4°C. After washing with PBS three times, cells were punched with 1% Triton and blocked with 2% horse serum for 1 hour at room temperature. Then, slides were incubated with primary antibody (LC3B antibody, 1 *μ*g/ml; NLRP3 antibody, 1/200 dilution; and CD68 antibody, 1/100 dilution, all from Abcam) at 4°C overnight. After being washed with PBS three times, samples were incubated with the corresponding IgG-FITC or Rhodamine-conjugated secondary antibody (dilution factor 1 : 200) for 2 h at room temperature. Cell nuclei were counterstained by DAPI (1 mg/ml) for 5 min. After a final wash, the slides were visualized under a laser scanning confocal microscope (Olympus FV1200, Olympus, Tokyo, Japan).

### 2.12. Statistical Analysis

All analyses were performed with SPSS 20.0 software (SPSS Inc., Chicago, IL, USA). Data are presented as the mean ± S.D. The multigroup comparisons were made with a one-way ANOVA analysis, followed by Dennett's post hoc test. Values of *P* < 0.05 were considered to indicate a statistically significant difference.

## 3. Results

### 3.1. Melatonin Inhibited Atherosclerotic Lesion Progression *In Vivo*

**Figure 1:**
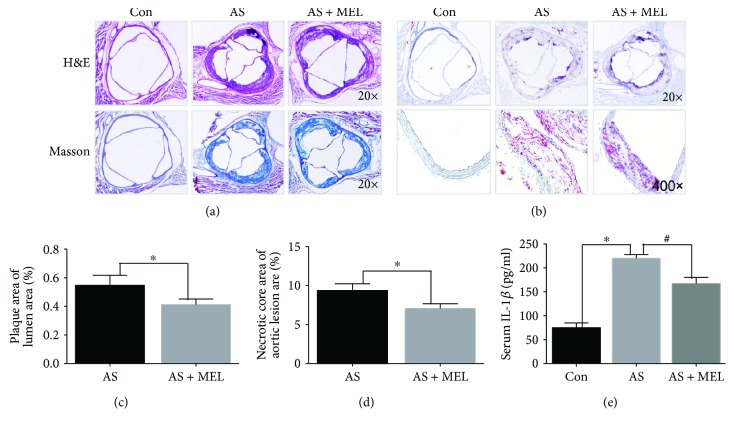
Melatonin inhibited atherosclerotic lesion progression *in vivo*. (a) Representative H&E and Masson staining images of atherosclerotic lesions of the aortic root area (*n* = 20). (b) Representative Oil Red staining images of atherosclerotic plaques of aortic root area (*n* = 20). (c) The atherosclerotic plaque size (represented as the ratio of plaque area/lumen area) in the melatonin-treated group as compared with the AS group (0.5769 ± 0.0780 versus 0.3836 ± 0.0340 %, *P* < 0.05). ^∗^ *P* < 0.05 versus the AS group. (d) The necrotic core area (represented as the ratio of necrotic core area/whole plaque area) in the melatonin-treated group as compared with the AS group (8.5569 ± 0.2780 versus 7.2636 ± 0.3340 %, *P* < 0.05). ^∗^ *P* < 0.05 versus the AS group. (e) The serum IL-1*β* level measured by the ELISA assay in the melatonin-treated group in comparison with the AS group (167.2 ± 12.7 versus 220.6 ± 8.79 pg/ml, *P* < 0.05). ^∗^ *P* < 0.05 versus the Con group; ^#^ *P* < 0.05 versus the AS group.

First, we examined the effect of melatonin on the atherosclerotic plaque progression. The AS model was successfully established, revealed by the plaque formation, and increased serum IL-1*β* level (75.3 ± 10.21 versus 220.6 ± 8.79 pg/ml, *P* < 0.05, Figure 1(e)) in Figures 1(a)–1(e). Figures 1(a) and 1(b) are the representative H&E, Masson, and Oil Red staining images for atherosclerotic plaques from sections across the aortic root. Quantification analysis revealed that melatonin treatment significantly reduced the plaque size as compared with the AS group (0.5769 ± 0.0780 versus 0.3836 ± 0.0340, *P* < 0.05, Figure 1(c)). Additionally, melatonin-treated mice showed a decreased necrotic core area (8.5569 ± 0.2780 versus 7.2636 ± 0.3340, *P* < 0.05, Figure 1(d)), indicating that melatonin decreased both the plaque size and the necrotic core. In agreement with plaque area, serum IL-1*β* level was markedly decreased by melatonin treatment in comparison with the AS group (167.2 ± 12.7 versus 220.6 ± 8.79 pg/ml, *P* < 0.05, Figure 1(e)). Taken together, the data demonstrated that melatonin inhibited plaque progression in the AS model.

### 3.2. Melatonin Inhibited NLRP3 Inflammasome Activation in Atherosclerotic Lesions

**Figure 2:**
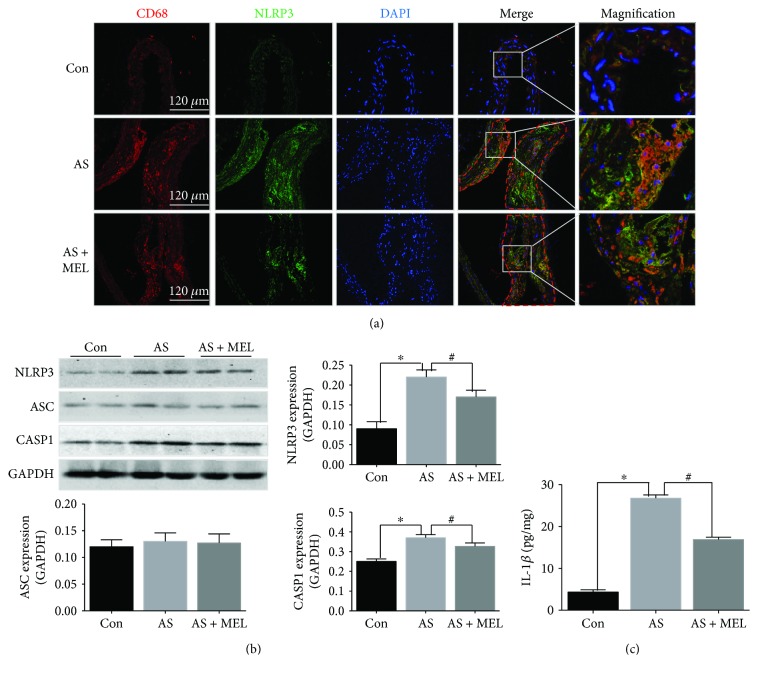
Melatonin inhibited NLRP3 inflammasome activation within atherosclerotic lesions. (a) The NLRP3 expression (the green color) and colocation with macrophage marker CD68 (the red color) in the aortic artery of melatonin treated mice as compared with AS mice (*n* = 20). (b) The NLRP3 and caspase-1 protein expression levels in atherosclerotic lesions as compared with the AS mice measured by Western blot (*n* = 20, *P* < 0.05). ^∗^ *P* < 0.05 versus the Con group; ^#^ *P* < 0.05 versus the AS group. (c) The IL-1*β* level measured by the ELISA assay within atherosclerotic lesions of melatonin treated AS mice in comparison with the AS group (*n* = 20, 16.89 ± 0.57 versus 26.77 ± 0.76 pg/mg, *P* < 0.05). ^∗^ *P* < 0.05 versus the Con group; ^#^ *P* < 0.05 versus the AS group.

Representative immunofluorescence staining images of NLRP3 and macrophage marker CD68 in AS lesion are presented in Figure 2(a). The CD68-positive staining area (in red color) shows the macrophage infiltration. In atherosclerotic plaques, CD68-positive macrophages were abundant around the lipid core area, indicating the significant macrophage infiltration within atherosclerotic lesions. Similarly, NLRP3-positive cells (in green color) were also found in this area, indicating the increased expression of NLRP3 in inflammatory AS plaque. Of note, NLRP3 staining was decreased in the aortic artery of melatonin-treated mice, suggesting that melatonin reduced the NLRP3 activation in atherosclerotic plaque. NLRP3 and consequent caspase-1 expressions by Western blot (Figure 2(b)) showed the same tendency, as melatonin markedly reduced NLRP3 and caspase-1 protein level in AS mice (*P* < 0.05). Furthermore, IL-1*β* level within lesions was markedly decreased by melatonin treatment in comparison with the AS group (16.89 ± 0.57 versus 26.77 ± 0.76 pg/mg, *P* < 0.05, Figure 2(c)).

### 3.3. Melatonin-Induced Sirt3 Activation and Mitophagy within Atherosclerotic Plaques

**Figure 3:**
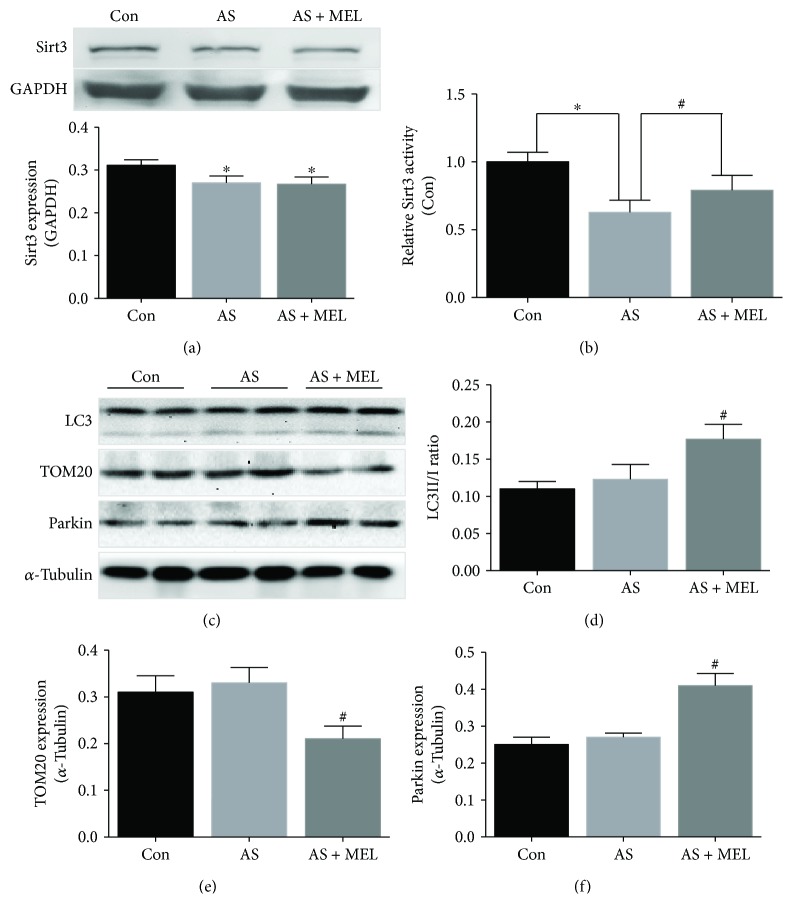
Melatonin induced Sirt3 activation and mitophagy in atherosclerotic plaques. (a) The Sirt3 expressions in the control group, AS group, and AS + MEL group were examined by Western blot assay (^∗^ *P* < 0.05 versus the Con group). (b) The Sirt3 activity in the melatonin-treated group in comparison with the AS group (*n* = 20, 0.894 ± 0.026 versus 0.635 ± 0.023, *P* < 0.05). ^#^ *P* < 0.05 versus the AS group. (c–f) The LC3II/I ratio, Parkin expression, and mitochondrial protein TOM20 level, which are the representative markers of the process of mitophagy, were examined by the Western blot assay (*n* = 20, *P* < 0.05). ^#^ *P* < 0.05 versus the AS group.

As shown by Western blot results in Figure 3(a), Sirt3 expression was slightly reduced in the AS group; however, there was no significant difference of Sirt3 expression between the AS and the AS + MEL groups (*P* > 0.05). Strikingly, Sirt3 activity was obviously elevated by melatonin (0.894 ± 0.026 versus 0.635 ± 0.023, *P* < 0.05, Figure 3(b)), indicating the protective role of melatonin was possibly dependent on Sirt3 activity. Mitophagy indexes including LC3, TOM20, and Parkin were also examined, as the elevated level of the LC3II/I ratio and Parkin expression, together with the reduced mitochondrial protein TOM20 expression, are well-recognized mitophagy markers. As is revealed by Western blot data in Figures 3(c)–3(f), melatonin significantly elevated the LC3II/I ratio and Parkin expression, while decreasing mitochondrial protein TOM20 level (*P* < 0.05), indicating that melatonin activated the process of mitophagy within atherosclerotic lesions.

### 3.4. Melatonin Suppressed Ox-LDL-Induced NLRP3 Inflammasome Activation in Macrophages

**Figure 4:**
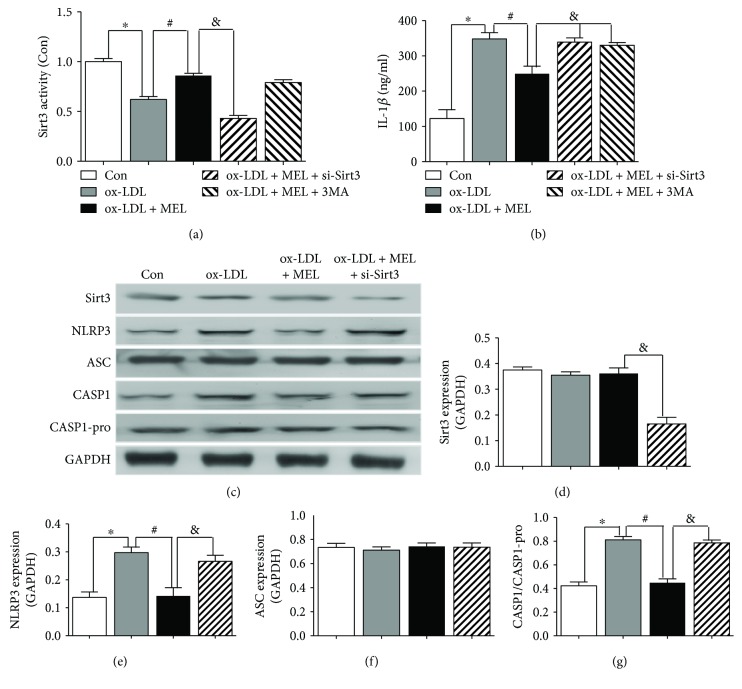
Melatonin suppressed ox-LDL-induced NLRP3 inflammasome activation in macrophages. (a) The Sirt3 activity examined by the ELISA assay. ^∗^ *P* < 0.05 versus the Con group; ^#^ *P* < 0.05 versus the ox-LDL group; and ^&^ *P* < 0.05 versus the ox-LDL + MEL group. (b) The IL-1*β* secretion level in macrophages examined by the ELISA assay. ^∗^ *P* < 0.05 versus the Con group; ^#^ *P* < 0.05 versus the ox-LDL group; and ^&^ *P* < 0.05 versus the ox-LDL + MEL group. (c–g) The Sirt3, NLRP3, ASC, caspase-1, and caspase-1-pro expressions were examined by the Western blot assay. ^∗^ *P* < 0.05 versus the Con group; ^#^ *P* < 0.05 versus the ox-LDL group; and ^&^ *P* < 0.05 versus the ox-LDL + MEL group.

Macrophage plays an essential role in the progression of AS. Therefore, we examined the effect of melatonin on inflammatory cytokine secretion and inflammasome activation *in vitro* by using ox-LDL-treated macrophages. To investigate the potential role of Sirt3 and mitophagy involved in the experimental condition, siRNA targeting Sirt3 and autophagy inhibitor 3-MA were applied. As was revealed in Figure 4(a), melatonin significantly diminished the ox-LDL-induced Sirt 3 activity reduction, and this effect was abrogated by siRNA targeting Sirt3. ELISA results (Figure 4(b)) showed that melatonin markedly reduced ox-LDL-induced IL-1*β* secretion in macrophages (249 ± 22.0 versus 348 ± 18.1 ng/ml, *P* < 0.05). Notably, both Sirt3 deletion by Sirt3-siRNA and autophagy inhibitor 3-MA abolished the beneficial effect of melatonin (339 ± 12.2 versus 249 ± 22.0 ng/ml and 330 ± 8.93 versus 249 ± 22.0 ng/ml, *P* < 0.05), suggesting that the function of melatonin was dependent on Sirt3 and the induction of mitophagy. NLRP3 inflammasome activation was explored by Western blot (Figures 4(c)–4(g)). Even though ASC expression was unchanged, NLRP3 expression was markedly increased in ox-LDL-treated cells, consequently elevating the activation of caspase-1 (indicated by increased caspase-1/caspase-1-pro ratio). Melatonin obviously decreased both NLRP3 and consequential caspase-1 activation, indicating that melatonin reduced ox-LDL-induced NLRP3 inflammasome activation. Both Sirt3 deletion by siRNA and autophagy inhibitor 3-MA partially reduced the effect of melatonin, demonstrating that the beneficial function of melatonin was mediated by Sirt3 and mitophagy.

### 3.5. Melatonin Inhibited NLRP3 Inflammasome Activation through Mitophagy Induction and ROS Scavenging

**Figure 5:**
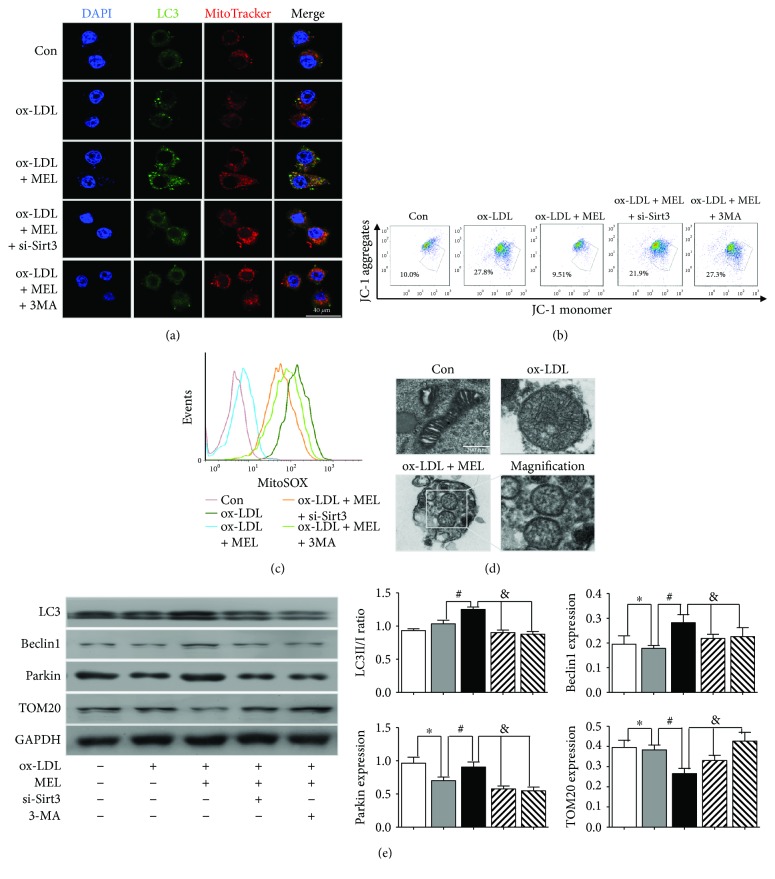
Melatonin inhibited NLRP3 inflammasome activation through mitophagy induction and ROS scavenging. (a) The colocalization of LC3 and MitoTracker in the melatonin-treated group was revealed by the immunofluorescence microscopy images. (b) The mitochondrial membrane potential (MMP) was examined by the JC-1 flow cytometry assay (ox-LDL group: 27.8%, ox-LDL + MEL group: 9.51%, ox-LDL + MEL + si-Sirt3 group: 21.9%, and ox-LDL + MEL+3MA group: 27.3%; *P* < 0.05). (c) The mitochondrial ROS generation in each group was revealed by the MitoSOX value results. (d) TEM images showed that induced mitochondrial impairment in macrophages, evidenced by swollen mitochondria with lysis of cristae. Moreover, the identifiable mitochondria in autophagosomes (the indicator for the presence of mitophagy) were observed in ox-LDL + MEL-grouped cells, confirming the presence of mitophagy. (e) The LC3II/I ratio, Beclin1, Parkin, and TOM20 expressions were examined by the Western blot assay. ^∗^ *P* < 0.05 versus the Con group; ^#^ *P* < 0.05 versus the ox-LDL group; and ^&^ *P* < 0.05 versus the ox-LDL + MEL group.

Since ROS is a recognized inducer for NLRP3 inflammasome activation, we further explored whether melatonin inhibited NLRP3 inflammasome activation through ROS scavenging. Immunofluorescence microscopy images (Figure 5(a)) revealed the induction of mitophagy by melatonin in ox-LDL-treated macrophages, evidenced by the overlapping of LC3 and MitoTracker (yellow dots in the ox-LDL + MEL group). Both Sirt3 deletion and autophagy inhibitor 3-MA decreased the effect of melatonin on mitophagy induction. The mitochondrial membrane potential (MMP) was monitored by JC-1 staining using the flow cytometry method (Figure 5(b)). Melatonin significantly reduced ox-LDL-induced MMP loss (9.51% versus 27.8%, *P* < 0.05). Of note, both Sirt3 deletion and 3-MA partially abolished the MMP recovery resulted from melatonin treatment (21.9% and 27.3% versus 9.51%, *P* < 0.05), indicating that the effect of melatonin depended on both Sirt3 and autophagy process. Consistent with MMP, melatonin reduced mitochondrial ROS generation in ox-LDL-treated macrophages, whereas either Sirt3-siRNA or 3-MA reversed the ROS scavenging caused by melatonin (Figure 5(c)). Additionally, mitochondrial impairment was also observed with a TEM micrograph (Figure 5(d)). The TEM microphage in the Con group shows the normal mitochondrial morphology, while ox-LDL stimulation induced swollen mitochondria with lysis of cristae which indicated the impairment of mitochondrial structures. Furthermore, the identifiable mitochondria in autophagosomes (the indicator for the presence of mitophagy) were observed in ox-LDL + MEL-grouped cells, reconfirming that melatonin induced mitophagy in ox-LDL-treated macrophages. Western blot results in Figure 5(e) revealed that melatonin induced the increase in the LC3II/I ratio and Beclin1/Parkin expression, while Sirt3-siRNA and 3-MA reversed the changes caused by melatonin. Taken together, these results demonstrated that melatonin caused mitophagy induction and ROS scavenging, which was mediated by Sirt3-dependent mitophagy.

### 3.6. Melatonin Activated Parkin-Mediated Mitophagy Process through the Sirt3-FOXO3a Pathway

**Figure 6:**
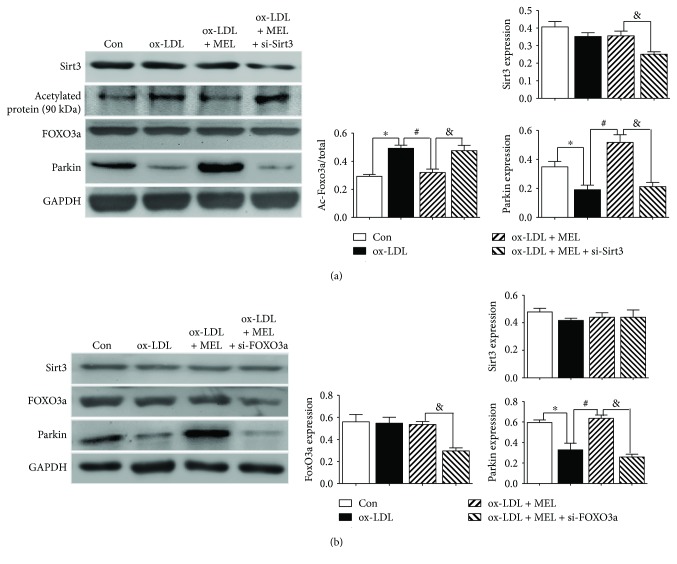
Melatonin activated Parkin-mediated mitophagy process through Sirt3-FOXO3a pathway. (a) The acetylation level of forkhead box O3a (FOXO3a) and the expression of Parkin were examined by the Western blot assay. ^∗^ *P* < 0.05 versus the Con group; ^#^ *P* < 0.05 versus the ox-LDL group; and ^&^ *P* < 0.05 versus the ox-LDL + MEL group. (b) The expressions of Sirt3, FOXO3a, and Parkin were examined by the Western blot assay. ^∗^ *P* < 0.05 versus the Con group; ^#^ *P* < 0.05 versus the ox-LDL group; and ^&^ *P* < 0.05 versus the ox-LDL + MEL group.

To explore the signaling pathway of melatonin in ox-LDL-treated macrophages, we highlighted mitophagy-related protein expressions using both Sirt3 and FOXO3 silencing by siRNA. As is demonstrated in Figure 6(a), melatonin promoted the deacetylation level of forkhead box O3 (FOXO3a, decreased ratio of acetylated protein of 90 kDa/total FOXO3a) and consequently increased the expression of Parkin. Sirt3 silence abolished the regulatory effect of melatonin on acetylated FOXO3a and Parkin. Similarly, FoxO3a deletion also reversed the alterations of the acetylated FOXO3a ratio and Parkin expression caused by melatonin (Figure 6(b)). Importantly, FOXO3a did not alter the expression level of Sirt3. Collectively, these mechanistic data revealed that the Sirt3-FOXO3a signaling pathway was associated with Parkin-mediated mitophagy.

## 4. Discussion

In this present study, we demonstrated, for the first time, that melatonin markedly inhibited AS plaque progression in a high fat dieted ApoE^−/−^ mice, mediated by mitophagy induction and decreased NLRP3 inflammasome activation. In ox-LDL-treated macrophages, melatonin reduced mitoROS level via the induction of mitophagy, consequently suppressing prolonged NLRP3 inflammasome activation. Furthermore, the beneficial effects of melatonin were associated with the Sirt3-FOXO3a-Parkin signaling pathway (schematic illustration in Supplementary Figure 1). Taken together, our data revealed the protective effect and underlying mechanism of melatonin on AS, which shed light on the therapeutic strategies of melatonin for clinical AS patients.

Melatonin, though mainly secreted by the pineal gland, is a widely produced indoleamine in all organisms [19]. The biological benefits of melatonin are mediated by several mechanisms, including antioxidant acting, reducing endoplasmic reticulum stress and DNA damage repair [20, 21]. Numerous researches have indicated that melatonin plays an important role in various cardiovascular diseases, such as myocardial ischemia-reperfusion injury, hypertension, abdominal aortic aneurysm, and cardiotoxicity induced by clinically used drugs [22–25]. Interestingly, a previous study using a HFD mouse model demonstrated that melatonin could reduce the plasma cholesterol level, indicating the beneficial role of melatonin against AS [26]. Furthermore, in a recent meta-analysis by Mohammadi-Sartang et al., they demonstrated that melatonin supplementation significantly improved triglycerides and total cholesterol levels in patients [27]. These studies indicated the protective role of melatonin against AS via the lipid profile regulation. However, the role of melatonin in atherosclerosis remains controversial. Even though several studies suggested that melatonin was reduced in AS plaque and inhibited atherosclerosis progression, controversies exist. For instance, Tailleux et al. reported that melatonin was highly increased in the surface of aortic atherosclerotic lesions and promoted atherosclerosis in the proximal aorta, but their results were based on the high melatonin dosage [11, 28, 29]. In our present study, we found that melatonin inhibited AS progression, through its anti-inflammatory properties.

The inflammatory nature of atherosclerosis has been established. Notably, searching for effective anti-inflammatory strategies for the treatment of AS underscores the importance of a better mechanistic understanding of atherosclerotic inflammation. Recently, the role of NLRP3 inflammasome in the disease of AS has drawn much attention [30, 31]. Inflammasomes are responsible for the conversion of proIL-1*β* and proIL-18 to mature inflammatory factors in response to danger signals such as pathogen-associated molecular patterns (PAMPs) and danger-associated molecular patterns (DAMPs) as intracellular complexes. In 2010, Duewell et al. first reported that NLRP3 inflammasomes are essential for high-fat diet-induced atherosclerosis [32]. It was also reported that NLRP3 inflammasome mRNA level was upregulated in a pig aorta in a diabetes mellitus-associated atherosclerosis [33]. Zheng et al. further demonstrated that silence of NLRP3 suppressed atherosclerosis and stabilized plaques in ApoE-deficient mice, indicating the prerequisite role of NLRP3 inflammasomes in the progression of atherosclerosis [34]. Moreover, it was recently confirmed that NLRP3 expression was significantly increased in human atherosclerotic plaques in comparison to nonatherosclerotic vessels [35]. Our current data was in agreement with these previous findings. NLRP3 inflammasome activation and inflammatory cytokine IL-1*β* secretion were upregulated in both AS plaque area and ox-LDL-treated macrophages. Strikingly, melatonin markedly inhibited NLRP3 inflammasome activation and IL-1*β* secretion, thus suppressing the progression of AS plaque. From these findings, we could conclude that melatonin exerted the protective effect against AS through the inhibition of NLRP3 inflammasome activation.

We next explored the underlying mechanisms by which melatonin attenuated NLRP3 inflammasome activation. Recent findings emphasized the mitochondria as a key regulator in inflammatory responses [36]. Specially, mitochondrial ROS have been suggested to be a critical activator of the NLRP3 inflammasome complex [37]. A previous research showed that mtROS overproduction by the inhibition of mitochondrial complex I or complex III triggered NLRP3 inflammasome activation [38, 39]. Similarly, the *in vitro* data demonstrated that ox-LDL stimuli attenuated MMP and increased mitoROS production in macrophages, consequently leading to NLRP3 inflammasome activation. Melatonin treatment in macrophages rescued MMP loss and decreased mitoROS generation, contributing to the alleviation of NLRP3 inflammasome activation. Mitophagy, mitochondria-selective autophagy, plays a central role in maintaining mitochondrial homeostasis through the elimination of a damaged mitochondria. The process of mitophagy is a balance regulator of NLRP3 inflammasome activation through mitoROS scavenging [40, 41]. Using both immunofluorescence staining and Western blot methods, we found that melatonin triggered mitophagy, which consequently attenuated mitoROS production and NLRP3 inflammasome activation. Furthermore, autophagy inhibitor 3-MA abolished the beneficial effects of melatonin on mitoROS scavenging, indicating that melatonin alleviated ox-LDL-induced mitoROS overgeneration and NLRP3 inflammasome activation was mitophagy dependent.

To date, the role of melatonin on Sirt3 has not been thoroughly investigated [42]. Sirt3 is a member of the class III deacetylases located in the mitochondria, regulating a variety of cellular biochemical processes by interacting with histone and nonhistone proteins [43, 44]. In the current study, we observed a decrease in Sirt3 expression in both AS plaque and ox-LDL-treated macrophages. With the method of Sirt3 silencing by using siRNA, we demonstrated that the protective role of melatonin against AS was mediated by Sirt3. Interestingly, melatonin elevated Sirt3 activity, without altering its expression level. The work of Pi et al. and Chen et al. provided the evidence regarding the likely involvement of Sirt3 in the hepatoprotective effect of melatonin [45, 46]. Zhai et al. recently reported that melatonin exerted cardioprotective effects by upregulating Sirt3 expression and activity [47]. This is contradictory with our data, since we only observed elevated Sirt3 activity but not expressed in the melatonin-treated group.

In the next step, the underlying signaling pathway was examined. As is evidenced by the Western blot results, deacetylation of FOXO3a and Parkin expression were decreased by ox-LDL stimuli, while melatonin reversed this decrease via Sirt3 activation. By using siRNA targeting Sirt3 and FOXO3a, we concluded that the regulatory effect of melatonin on Parkin-mediated mitophagy was Sirt3/FOXO3a dependent. In a recent work by Díaz-Casado et al., they demonstrated that melatonin restored the Parkin/Pink1 network and rescued mitochondrial function in a zebrafish Parkinson's disease model [48]. Similarly, Yu et al. previously reported the involvement of Sirt3/FOXO3a/Parkin-mediated mitophagy in diabetic cardiac dysfunction [49]. Herein, we demonstrated for the first time the protective effects of melatonin on Sirt3/FoxO3a/Parkin-mediated mitophagy in ox-LDL-stimulated macrophages.

Despite the potential clinical relevance of our findings, there are some limitations in the present study. Even though we applied Sirt3-siRNA to demonstrate the beneficial role of melatonin was Sirt3 dependent in macrophages, *in vivo* evidence was limited due to the lack of Sirt3 knockout mice. Secondly, our current research has not elucidated the possibility that melatonin might have a direct effect on ox-LDL and which melatonin receptor is responsible for Sirt3 activation. Thirdly, the plasma cholesterol levels were not examined in the present study, which might be a potential contributor to the beneficial effect of melatonin. These above-mentioned limitations need further investigation in a future work.

Collectively, our study revealed that melatonin prevented atherosclerotic progression via the blockage of NLRP3 inflammasome activation and inflammatory factor secretion, which was a novel mechanism of melatonin against AS. Melatonin induced Sirt3/FOXO3/Parkin-mediated mitophagy and decreased mitochondrial ROS production, thereby attenuating NLRP3 inflammasome activation in macrophages. Taken together, our study provides novel insight into melatonin as a new target for therapeutic intervention for AS.

## Data Availability

The data used to support the findings of this study are available from the corresponding author upon request.

## Supplementary Materials

Supplementary Materials The schematic illustration of the main findings was provided with a graphical abstract in Supplementary Figure 1.
