# Supplementary material for: Melatonin Ameliorates the Progression of Atherosclerosis via Mitophagy Activation and NLRP3 Inflammasome Inhibition
Source: Oxid Med Cell Longev. 2018 Sep 4;2018:9286458. doi: 10.1155/2018/9286458 (PMC6142770; doi:10.1155/2018/9286458)
Supplement: Supplementary Materials — The schematic illustration of the main findings was provided with a graphical abstract in Supplementary Figure 1. [file 9286458.f1.pdf]

Graphical Abstract

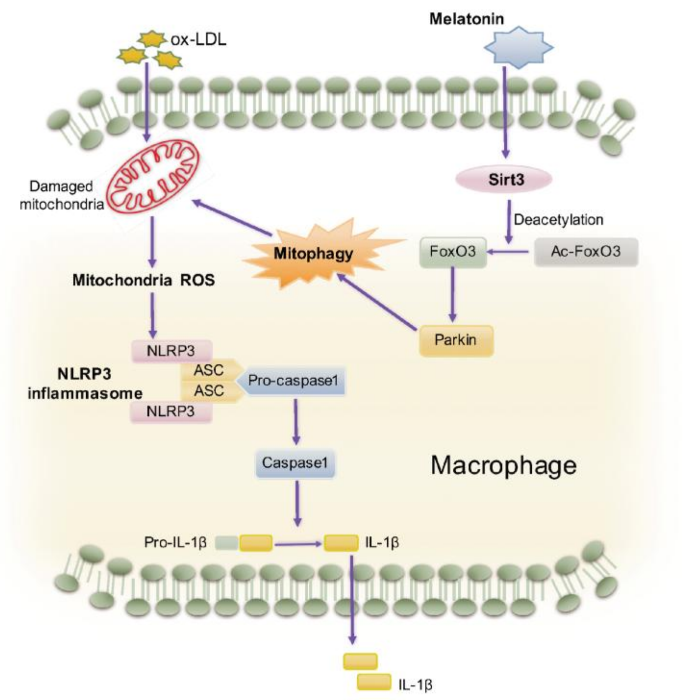

Ox-LDL induced mitochondrial damage, which contributed to the excessive mitoROS generation and consequential NLRP3 inflammasome activation in macrophages. Melatonin activated Sirt3 and FoxO3/Parkin-mediated mitophagy, attenuated the mitoROS level and the NLRP3 inflammasome activation, and subsequently reduced Caspase 1 activation and IL-1β secretion.
